# The Potential Role of CDH1 as an Oncogene Combined With Related miRNAs and Their Diagnostic Value in Breast Cancer

**DOI:** 10.3389/fendo.2022.916469

**Published:** 2022-06-16

**Authors:** Dan Xie, Yiyu Chen, Xue Wan, Jingyuan Li, Qin Pei, Yanan Luo, Jinbo Liu, Ting Ye

**Affiliations:** Department of Laboratory Medicine, The Affiliated Hospital of Southwest Medical University, Sichuan, China

**Keywords:** CDH1, miRNAs, oncogene, breast cancer, biomarker

## Abstract

**Background:**

Breast cancer (BC) is the leading cause of cancer−related mortality in females and the most common malignancy with high morbidity worldwide. It is imperative to develop new biomarkers and therapeutic targets for early diagnosis and effective treatment in BC.

**Methods:**

We revealed the oncogene function of cadherin 1 (CDH1) *via* bioinformatic analysis in BC. Moreover, miRNA database was utilized to predict miRNAs upstream of CDH1. Expression of CDH1-related miRNAs in BC and their values in BC stemness and prognosis were analyzed through TCGA‐BRCA datasets. In addition, Gene Ontology (GO) and Gene Set Enrichment Analysis (GSEA) were performed to explore the potential functions and signaling pathways of CDH1 in combination with CDH1-related miRNAs in BC progression. Finally, the differential expressions of soluble E-cadherin (sE-cad), which is formed by the secretion of CDH1-encoded E-cadherin into serum, analyzed by enzyme-linked immunosorbent assay (ELISA). Reverse transcription quantitative real-time polymerase chain reaction (RT-qPCR) was used to detect the expression level of CDH1-related miRNAs in serum samples.

**Results:**

The mRNA and protein expressions of CDH1 were elevated in BC tissues compared with normal counterparts. Moreover, CDH1 overexpression was positively correlated with BC stage, metastatic, stemness characteristics, and poor prognosis among patients. In predictive analysis, miR-340, miR-185, and miR-20a target CDH1 and are highly expressed in BC. miR-20a overexpression alone was strongly associated with high stemness characteristics and poor prognosis of BC. Additionally, GO, KEGG, and hallmark effect gene set analysis demonstrated that CDH1 in combination with overexpression of miR-340, miR-185, or miR-20a participated in multiple biological processes and underly signaling pathways involving in tumorigenesis and development of BC. Finally, we provide experimental evidence that the combined determination of serum sE-cad and miR-20a in BC has highly diagnostic efficiency.

**Conclusions:**

This study provides evidence for CDH1 as an oncogene in BC and suggests that miR-20a may regulate the stemness characteristics of BC to exert a pro-oncogenic effect by regulating CDH1. Moreover, sE-cad and miR-20a in serum can both be used as valid noninvasive markers for BC diagnosis.

## Introduction

Breast cancer (BC) is the leading cause of cancer−related mortality in females and the most common malignancy with high morbidity worldwide (1). Despite of diagnosis and therapeutic predominant advancements of BC, its treatment efficacy and prognosis have not been substantially improved, generally attributed to late diagnosis and tumor metastasis (2, 3). Therefore, it is imperative to develop new biomarkers and therapeutic targets for early diagnosis and effective treatment in BC. Furthermore, a growing body of evidence indicates that cancer stem cells (CSCs, also known as tumor-initiating cells or tumor-propagating cells) are a small subpopulation of cells with self-renewal capability and differentiation potential, which are considered to be the root of cancer initiation, metastasis, treatment resistance, and poor prognosis (4, 5). Therefore, identifying gene signatures associated with the characteristics of CSCs has diagnostic and prognostic implications.

The CDH1 gene encodes E-cadherin (E-cad) protein, a calcium-dependent transmembrane adhesion that is crucial for maintaining pluripotency and self-renewal of embryonic stem cells and neural stem cells (6–8). CDH1 is commonly reported as a tumor suppressor gene in cancer literature (9). Down-regulation or loss of E-cad encoded by CDH1 is also known to contribute to malignant tumor invasion and metastasis (10–12). Recent studies have demonstrated that CDH1 and its encoded E-cad have oncogenic properties. CDH1 oncogene, for example, induces self-renewal of lung cancer stem-like cells (13). E-cad^+^ subsets of prostate cancer cells displayed characteristics associated with cancer stem cells, and prostate cancer stem cells exhibit the plasticity of E-cad expression during cell invasion (14, 15). In BC, high expression of E-cad in SKBR3 cells was allowed to enhance mammosphere formation (16). Moreover, Padmanaban et al. (17) demonstrated that E-cad promotes metastasis in murine and human models of both luminal and basal-like breast cancer. In addition to above observations, a reported study has revealed that even though E-cad is a transmembrane molecule, its extracellular structure can be cleaved off and released into the bloodstream in the soluble form, also known as soluble E-cadherin (sE-cad) (18). Numerous publications have discussed sE-cad, which is highly expressed in the serum of patients with malignant tumors, as a diagnostic and prognostic biomarker of malignancy (19–22). The serum level of sE-cad in lung cancer patients was significantly higher than in control subjects, and patients with distant metastasis had an even more significant increase (21). The high serum sE-cad level was also found to positively correlate with TNM stage, tumor grade, and lymph node metastasis in BC (22). These results demonstrated that the tumor suppressor or pro-oncogene role of CDH1 in malignant tumors is controversial and has not been well elucidated.

MicroRNAs (miRNAs) are noncoding RNAs that regulate gene expression by identifying cognate sequences and interfering with transcription, translation, and epigenetic processes (23). Several miRNAs can be oncogenes or tumor suppressors, and their dysregulation leads to cancer initiation, progression, and metastasis (24).Furthermore, miRNAs can be secreted into the bloodstream and remain highly stable in serum or plasma (25). As a result, circulating miRNAs are considered ideal tumor biomarkers. miRNAs in serum or plasma are increasingly recognized as molecular markers for non-invasive diagnosis and prognosis of cancer (26). For example, serum miR-103a-3p could serve as a potential non-invasive diagnostic and prognostic biomarker for BC (27). In prostate cancer, the higher levels of miR-1290 and miR-375 were significantly associated with poorer overall survival (28).

Bioinformatics analysis of Oncomine, TIMER, TCGA, GEO and GEPIA databases in the present study revealed that CDH1 may function not only as a tumor suppressor but also as a pro-oncogene capable of accelerating the malignant progression of BC. In predictive analysis, miR-340, miR-185, and miR-20a target CDH1 and are highly expressed in BC. GO, KEGG and hallmark gene set analyzes were then used to investigate the potential functions and signaling pathways of CDH1 in combination with overexpression of miR-340, miR-185, or miR-20a in BC. This suggests that these miRNAs could regulate CDH1’s oncogenic mechanisms. Finally, we provide experimental evidence that combined determination of serum sE-cad and miR-20a in BC has high diagnostic efficiency. The results of this study reveal a novel role for CDH1 and miRNAs that regulate it as BC pro-oncogenes, and suggest that serum sE-cad and miR-20a are potentially noninvasive diagnostic markers for BC.

## Materials and Methods

### Oncomine Analysis

Oncomine (http://www.oncomine.org/), a web-based cancer microarray database, is used to compare the transcriptome data in most major types of cancer with respective normal tissues (29). The gene expression levels of CDH1 in BC was identified in Oncomine.

### TIMER2.0 Database Analysis

TIMER2.0 (http://timer.comp-genomics.org/) is a tumor related database (30). Timer algorithms to learn about the differences of CDH1 expressions was performed using TIMER2.0.

### The Cancer Genome Atlas (TCGA) Dataset Analysis

Gene expression profile for BC cancer patients was obtained from TCGA data portal (https://portal.gdc.cancer.gov/) (31). Clinical data such as gender, age, histological type, and survival were also downloaded from TCGA data portal. The original data from TCGA was normalized and analyzed by R language.

### GEO Datasets Selecting and Differential Analysis

The two gene expression datasets of BC from GEO database were downloaded, including the following criterias: (a) BC, (b) datasets including tumor and normal tissues, (c) the organism is *Homo sapiens*, (d) sample size exceeding 30 samples. GSE45255 and GSE2603 were among depended on the GPL90 platform (32). The limma package was used to identify the DEGs in each GEO datasets in R. The *P*-value is determined by the false discovery rate.

### Gene Expression Profiling Interactive Analysis (GEPIA) Database Analysis

The online database GEPIA (http://gepia2.cancer-pku.cn/index) is a web-based database that includes 9,736 tumors and 8,587 normal samples, which analyze the RNA sequencing expression between the tumor and normal tissue (33). GEPIA was used to further confirm the differential expression of genes.

### Human Protein Atlas Analysis (HPA) Analysis

HPA (https://www.Proteinatlas.org/) makes use of antibody method for immunostaining on differential expression analysis of proteins in normal and tumor tissues (34). We checked the expression of CDH1 in the protein expression module of the HPA database and analyzed the immunohistochemical results of CDH1 in tumor tissues and normal tissues (Antibody: CAB072856).

### Immunohistochemical Staining

The paraffin-embedded tissues were collected from the Pathology Department of the Affiliated Hospital of Southwest Medical University. The tissue slides were then deparaffinized, rehydrated, and stained overnight at 4°C with a 1:500 dilution of the rabbit polyclonal E-cadherin antibody (208741AP, Proteintech). Moreover, the slides were incubated with streptavidin horseradish peroxidase (HRP) after being treated with biotinylated secondary antibody. Finally, they were stained with 3, 3’-Diaminobenzidine (DAB) and haematoxylin counterstained. Immunostaining intensity was graded as follows: 1 mild (+), 2 moderate (++), and 3 high (+++).

### Kaplan-Meier Plotter Database

The prognostic significance of mRNA expression in BC cancer was evaluated using the Kaplan-Meier plotter (www.kmplot.com) (35). The overall survival (OS), distant metastasis-free survival (DMFS), and recurrence-free survival (RFS) of BC cancer patients were analyzed by the Kaplan-Meier survival plot.

### Calculation of the Gene Expression-Based Stemness Index (mRNAsi)

The one-class logistic regression (OCLR) algorithm was used to calculate the stemness index based on gene expression profiles of normal PSCs (36). The stemness signature was generated with the OCLR algorithm by utilizing the gelnet package in R. Then, we calculated the Spearman correlations between the weight vectors of the stemness signature and mRNA expressions of BC samples. The stemness index generated from gene expression profiles was defined as mRNAsi.

### Pearson’s Correlation Analysis

The co-expression analysis was constructed using R to show Pearson correlation coefficient between two genes.

### Gene Set Enrichment Analysis (GSEA)

GSEA was carried out between datasets with high or low CDH1 and mircro-RNAs mRNA expression (37). The Hallmark effector gene sets and the Kyoto Encyclopedia of Genes and Genomes (KEGG) signaling pathway associated with CDH1 and mircro-RNAs mRNA expression were annotated. GSEA software was obtained from the Broad Institute (http://www.broad.mit.edu/gsea).

### Analyses of miRNA-mRNA Targets

In order to predict miRNAs upstream of CDH1, we used the StarBase (http://starbase.sysu.edu.cn/), miRDB (http://www.mirdb.org/), miRWalk (http://mirwalk.umm.uni-heidelberg.de/), microRNA (www.microrna.org/), and Targetscan (http://www.targetscan.org/) to locate the potential miRNAs targeting CDH1.

### The Analysis of Diagnostic Efficiency

The receiver operating characteristic (ROC) curve was used to illustrate the diagnostic efficiency of CDH1, microRNAs, and sE-cad.

### Clinical Samples

The patient’s consent was obtained according to the protocol approved by the Institutional Review Committee of the Affiliated Hospital of Southwest Medical University. All samples were obtained from the Department of Medical Laboratory, Affiliated Hospital of Southwest Medical University. A total of 100 serum samples were collected, including 50 healthy controls and 50 patients with pathological diagnosis of BC. Each patient with BC was classified according to AJCC Cancer Staging Manual.

### Isolation of miRNAs From Serum

According to the manufacturer’s protocol, total RNA was extracted from serum (volume, 200 µl) using a miRcute miRNA extraction isolation kit (DP501, Tiangen, China). These RNAs were then reverse-transcribed into cDNA using the instructions of All-in-OneTM miRNA qRT-PCR kit (GeneCopoeia, the US).

### RT-qPCR Analysis

All-in-one miRNA qRT-PCR kit (GeneCopoeia, the US) was used to perform RT-qPCR according to the manufacturer’s protocol, and primers used in experiments were as follows:

miR-340:5′- CTTATAAAGCAATGAGACTGATTAAA-3′;
miR-185:5′-GAGAGAAAGGCAGTTCCTGAAA-3′;
miR-20a:5′- TAAAGTGCTTATAGTGCAGGTAGAA-3′;

Cel-miR-39 was used as an external reference control in serum. Previous to RNA extraction, 1 µL of cel-miR-39 (GeneCopoeia, the US) at 2 µM was added as spike-in exogenous control and used as exogenous control for data normalization. Each sample was measured in triplicate. The relative expression of the mRNA was calculated using ΔCq, and the fold change was calculated using the 2 -ΔΔCq method.

### ELISA Analysis

Using a commercial ELISA kit (Ruixin Biological Technology Co., Ltd, Quanzhou, China) following the manufacturer’s instructions, sE-cad expression was tested in human serum. The optical density (OD) was measured at 450 nm, the standard curve was established with OD 450 as Y-axis, and the reference substance concentration as X-axis and protein expression was obtained by standard curve.

### Statistical Analysis

The differential expression of sE-cad and miRNAs in serum was analyzed using Student’s t test. Data were analyzed using GraphPad Prism 8 software, and *P* < 0.05 was considered statistically significant.

## Results

### High Expression of CDH1 in BC Patients

**Figure 1:**
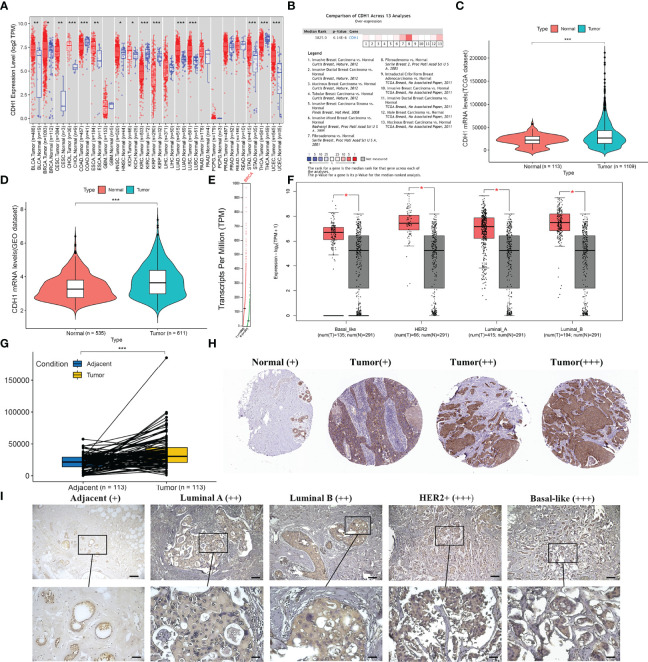
CDH1 expression is increased in human breast cancer tissues. **(A)** CDH1 gene expression profile across 21 types of tumor samples and normal tissues from TIMER2.0 analysis. **(B)** Oncomine analysis of CDH1 expression in human cancers. **(C-E)** CDH1 expression in TCGA, GEO, and TIMER2.0 is higher in BC tissues than in normal tissues. **(F)** GEPIA database analysis of CDH1 expression was increased in all subtypes of BC tissues compared to normal tissue. **(G)** TCGA database shows higher levels of CDH1 expression in BC tissues than in pericarcinomatous tissues. **(H)** HPA database shows higher expression of E-cadherin in BC tissues than in normal tissues. **(I)** Representative immunohistochemical staining of E-cadherin in multiple BC subtypes. Scale bar = 60μm (upper panels), Scale bar = 30μm (lower panels). *P < 0.05; **P < 0.01; ***P < 0.001.

We first compared the mRNA levels of CDH1 in 21 types of cancers with their normal counterparts by using TIMER2.0 database. CDH1 mRNA expression was increased in 11 types of cancers, decreased in 4 types of cancers, and there was no significant difference in 6 types of cancers (**Figure 1A**). These findings revealed that increased mRNA expression of CDH1 is a rather common feature of human cancer that currently merits highly appreciated. Meanwhile, we conducted in-depth analysis in BC by other public databases. We initially analyzed CDH1 transcription levels in BC tissues in Oncomine and found that CDH1 was expressed at a higher level in BC tissues compared to normal tissues through 13 datasets (**Figure 1B**; *P* = 6.14e-06). We then conducted CDH1 expression analysis using TCGA-BRCA, GEO datasets, and TIMER2.0. Similarly, CDH1 was significantly overexpressed in BC tissues compared to normal tissues (**Figures 1C–E**). As BC is a highly heterogeneous malignant neoplasm, we investigated CDH1 expression in different subtypes of BC in GEPIA. CDH1 expression is significantly overexpressed in each BC subtype (**Figure 1F**). TCGA-BRCA also indicated that CDH1 expression in BC tissues were was considerably higher than those in adjacent tissues (**Figure 1G**). To investigate the correlation between mRNA and protein expression, we examined the differences in CDH1 protein expression between tumor and normal tissues by using Human Protein Atlas Analysis (HPA) database. The HPA database indicated that the protein expression of CDH1 in BC tumor tissues was significantly higher than that in normal tissues (**Figure 1H**). Moreover, to verify the findings of the HPA database, we detected the protein expression of CDH1 (E-cadherin) with immunohistochemical staining (IHC). The paraffin-embedded tissues were collected for CDH1 protein analysis, including different subtypes BC cases (Luminal A, Luminal B, HER2+ and TNBC) and their matched adjacent normal tissues. What can be clearly seen in immunohistochemical figures is that the E-cadherin expression was significantly up-regulated in BC tissues compared with corresponding adjacent normal tissues (**Figure 1I**). These results indicate that CDH1 is overexpressed in BC tissues.

### Correlation Analysis Between CDH1 Overexpression and Malignant Progression in BC Patients

**Figure 2:**
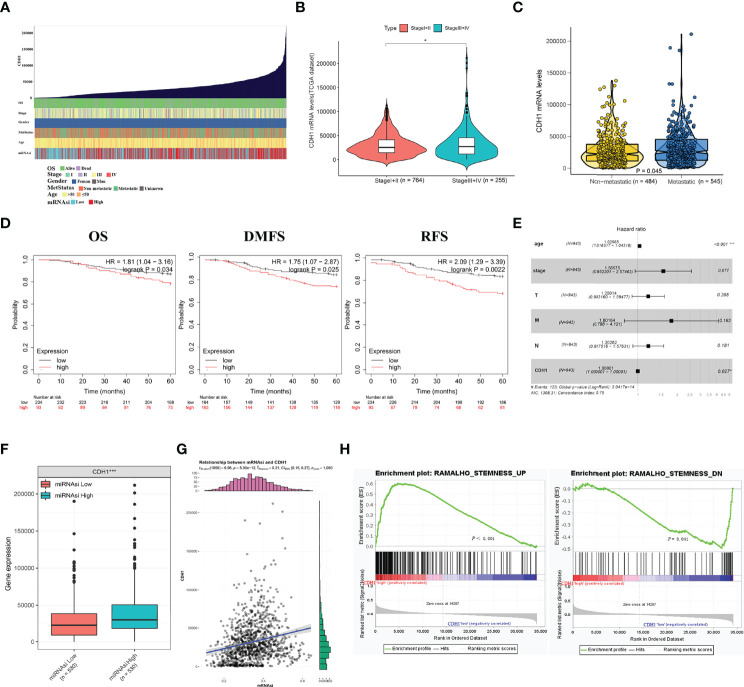
CDH1 overexpression is intimately associated with the malignant progression of breast cancer. **(A)** An overview of the association between CDH1 and clinical features and stemness in BC. **(B)** Differential expressions of CDH1 in I+II and III+IV tumor stage in BC. **(C)** Differential expressions of CDH1 in non-metastatic and metastatic BC. **(D)** Elevated expression of CDH1 indicated poor clinical outcomes for BC patients were plotted from Kaplan-Meier plotter database. **(E)** Multivariate analysis of the correlation of CDH1 expression with OS among BC patients. **(F)** TCGA database analysis of CDH1 expression differences in BC mRNAsi high and low groups. **(G)** Correlation of CDH1 expression and mRNAsi in BC. **(H)** GSEA assessment of the enrichment score profile of CDH1 expression in the stemness high and low groups. OS, overall survival; RFS, relapse-free survival; DMFS, distant metastasis-free survival. *P < 0.05; ***P < 0.001.

We explored the associations between CDH1 and clinical features and stemness in TCGA-BRCA samples (**Figure 2A**). As shown in **Figure 2B**, CDH1 expression in patients with advanced stages (III-IV) was significantly higher than in those with early stages (I-II). Likewise, the markedly differences occurred between the metastatic and non-metastatic patients (**Figure 2C**). Then, the Kaplan-Meier plotter tool was used to explore the correlation between CDH1 expression and clinical outcomes in BC. BC patients with CDH1 overexpression showed worse overall survival (OS) [HR = 1.81, *P* = 0.034], distant metastasis-free survival (DMFS) [HR = 1.75, *P* = 0.025] and relapse-free survival (RFS) [HR = 2.09, *P* = 0.0022] than patients with minimal expression of CDH1 (**Figure 2D**). Cox multivariate analysis revealed that CDH1 high expression was an independent risk factor for a poor prognosis in BC patients (**Figure 2E**; *P* = 0.027). Afterwards, the relationships between the mRNA expression of CDH1 and clinicopathologic features in BC are summarized in **Table 1**. High CDH1 expression was positively associated with Metastasis Status (*P* = 0.014), ER (*P* = 0.019), and PR (*P* = 0.003) expressions. In addition, Malta et al. (36) used one-class logistic regression (OCLR) to generate a stemness index for evaluating the dedifferentiation degree of cancer and proposed a stemness index mRNAsi based on mRNA expression. The high value of mRNAsi was positively correlated with active biological processes in CSCs and tumor dedifferentiation. Therefore, we further evaluated the relationship between the stemness index and CDH1 expression in BC. The results showed that the CDH1 expression was significantly higher in high mRNAsi group than in low mRNAsi group of BC (**Figure 2F**), and high CDH1 expression was associated with higher mRNAsi (**Figure 2G**). Meanwhile, GSEA enrichment analysis revealed that CDH1 overexpression was positively correlated with stemness-related signatures (**Figure 2H**). These results suggest that CDH1 overexpression is positively associated with stemness in BC. CDH1 not only mediates the cancer progression but also serves as a risk factor and predictor of poor prognosis in BC.

**Table 1:** Correlations between CDH1 expression in tissue and clinicopathological parameters of BC patients.

Variables	All cases	CDH1 mRNA		*p*-value
		Low (n=352)	High (n=352)	
**Age**				
< 51	208	101	107	0.62
≥ 51	496	251	245	
**Stage**				
I+II	530	261	269	0.471
III+IV	174	91	83	
**Lymphoid Nodal Status**				
Negative	347	185	162	0.083
Positive	357	167	190	
**Metastasis Status**				
Negative	698	352	346	0.014*
Positive	6	0	6	
**ER**				
Negative	158	92	66	0.019*
Positive	546	260	286	
**PR**				
Negative	235	120	115	0.65
Positive	469	232	237	
**HER2**				
Negative	545	289	256	0.003*
Positive	159	63	96	

*Significantly different.

ER, estrogen receptor; PR, progesterone receptor; HER2, human epidermal growth factor receptor 2; Ki-67, proliferating antigen Ki67.

### Expression of miRNAs-Targeted CDH1 and Prognostic Significance

**Figure 3:**
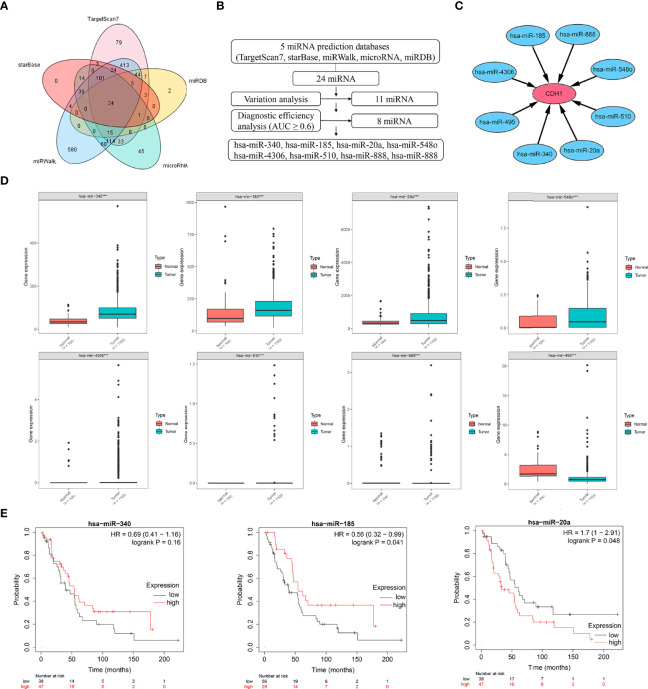
Expression and prognostic significance of miRNAs targeting CDH1. **(A)** Venn diagram of potential miRNAs predicted to regulate CDH1 from a total of five databases: Targetscan7, miRDB, microRNA, miRWalk, and starBase. **(B, C)** Expression and diagnostic efficiency analysis of miRNAs regulating CDH1. **(D)** TCGA database analysis of gene expression of miRNAs regulating CDH1. **(E)** Kaplan-Meier plotter database analysis of the significance of miR-340, miR-185, and miR-20a expression in breast cancer prognosis. ***P < 0.001.

miRNAs have been identified as critical regulators of gene expression. As a result, we used Targetscan7, miRDB, microRNA, miRWalk, and starBase to predict potential miRNAs that regulate CDH1, and from Venn diagram, we obtained 24 miRNAs that may regulate CDH1 (**Figure 3A**). Through difference analysis and diagnostic efficiency (AUC ≥ 0.6) screening, eight significant differential expression miRNAs, respectively miR-340, miR-185, miR-20a, miR-548o, miR-4306, miR-510, miR-888, and miR-495 were identified (**Figures 3B, C**). Then, the expression degree of eight miRNAs regulating CDH1 was analyzed in BC, and the results showed that miR-340, has-miR-185, and miR-20a had overexpression in BC tissues, while miR-548o, miR-4306, miR-510, miR-888 and miR-495 are lower in BC (**Figure 3D**). Therefore, we identified miR-340, miR-185, and miR-20a as candidates for further investigation and validation. Subsequently, Kaplan-Meier analysis revealed that miR-340 expression had no correlation with OS in BC patients [HR =0.69, *P* = 0.16], and miR-185 low expression was correlated with poor OS in BC patients [HR = 0.56, *P* = 0.041]. High expression of miR-20a was correlated with poor OS in BC patients [HR = 1.7, *P* = 0.048] (**Figure 3E**).

### Correlation Between miR-340, miR-185 and miR-20a and Stemness Features of BC

**Figure 4:**
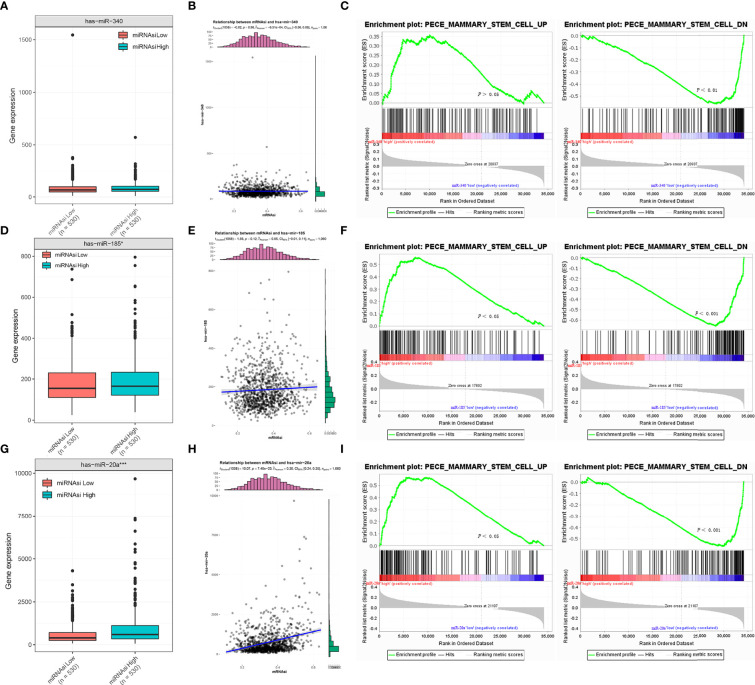
Correlation analysis between miR-340, miR-185, and miR-20a and stemness signature of breast cancer. **(A, D, G)** TCGA database analysis of miR-340, miR-185 and miR-20a expression differences in BC mRNAsi high and low groups. **(B, E, H)** Association analysis between miR-340, miR-185 and miR-20a expression and mRNAsi in BC. **(C, F, I)** GSEA assessment of the enrichment score profile of miR-340, miR-185, and miR-20a expression in the stemness high and low groups. *P < 0.05; ***P < 0.001.

We performed expression analysis and enrichment analysis to explore the relationship between the expressions of miR-340, miR-185, miR-20a, and the stemness features of BC. The results showed no differences in the expression of miR-340 between the high mRNAsi group and the low mRNAsi group in BC (**Figure 4A**), and no association between miR-340 expression and mRNAsi (**Figure 4B**). The enrichment analysis indicated that miR-340 expression had no significant correlation with the stemness characteristics in BC (**Figure 4C**). The expressions of miR-185 and miR-20a in the high mRNAsi group of BC were significantly higher than those in the low mRNAsi group (**Figures 4D, G**). However, we found that only an association of high miR-20a expression with higher mRNAsi (**Figure 4H**), whereas miR-185 expression was not correlated with mRNAsi (**Figure 4E**). The enrichment analysis also revealed that the increased expressions of miR-185 and miR-20a were positively correlated with the stemness characteristics of BC (**Figures 4F, I**). These findings show that the overexpression of miR-185 and miR-20a, which target CDH1, promotes the stemness characteristics and is also linked to the malignant progression of BC.

### Functional Enrichment Analysis of CDH1 Combination With miR-340, miR-185, miR-20a

**Figure 5:**
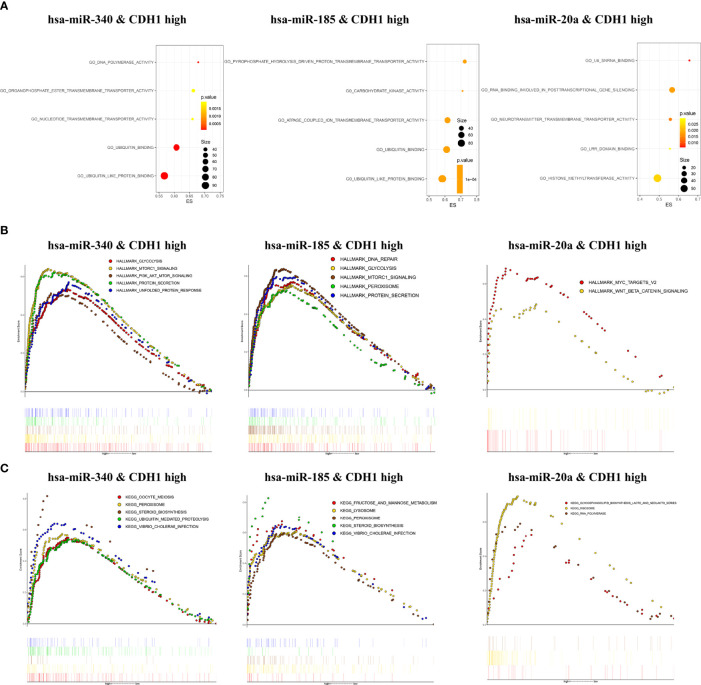
Enrichment analysis of CDH1 in combination with miR-340, miR-185, or miR-20a. **(A)** Gene Ontology functions enrichment analysis. **(B)** Hallmark gene set enrichment analysis. **(C)** KEGG pathways enrichment analysis.

To investigate the biological functions and regulatory mechanisms of CDH1 in combination with miR-340, miR-185, or miR-20a in BC, we performed functional enrichment analysis in the TCGA BC samples. GO enrichment analysis showed that CDH1 overexpression combined with miR-340 was highly _enriched in ubiquitin-like protein binding, ubiquitin binding, nucleotide transmembrane transporter activity, organophosphate ester transmembrane transporter activity, and DNA polymerase activity. On the other hand, the high expression of CDH1 in combination with miR-185 is over-increased in ubiquitin-like protein binding, ubiquitin-binding, ATPase coupled ion transmembrane transporter activity, pyrophosphate hydrolysis-driven proton transmembrane transporter activity, and carbohydrate kinase activity. The high expression of CDH1 in combination with miR-20a is highly enriched in histone methyltransferase activity, RNA involved in post-transcriptional gene silencing, LRR domain binding, neurotransmitter transmembrane transporter activity, and stem cells U6 SNRNA binding (**Figure 5A**). Furthermore, GSEA was used to assess the signaling pathways associated with the hallmark effector gene and KEGG signaling pathway. CDH1 overexpression combined with miR-340 in the Hallmark gene is abundant in MTORC1 signaling, protein secretion, unfolded protein response, glycolysis and PI3K/AKT/MTOR signaling. CDH1 combined with miR-185 is enriched in MTORC1 signaling, protein secretion, DNA repair, glycolysis, and peroxisomes. However, the MYC targets V2 and WNT/BETA-CATENIN signaling were the most significant enrichment for the highly expression of CDH1 combined with miR-20a (**Figure 5B**). In KEGG, CDH1 combined with miR-340 overexpression was highly enriched in steroid biosynthesis, vibrio cholerae infection, peroxidase, oocyte meiosis, and ubiquitin-mediated proteolysis. CDH1 combined with miR-185 overexpression was enriched primarily in steroid biosynthesis, vibrio cholerae infection, lysosomes, peroxidase, fructose, and mannose metabolism. Concurrently high expression of CDH1 combined with miR-20a was mainly enriched in ribosomal, RNA polymerase, glycosphingolipid biosynthesis lacto, and neolacto series (**Figure 5C**).

### CDH1 Encoded E-Cad Secreted Serum sE-Cad Combined With miR-20a Has Good Diagnostic Potential in BC Patients

**Figure 6:**
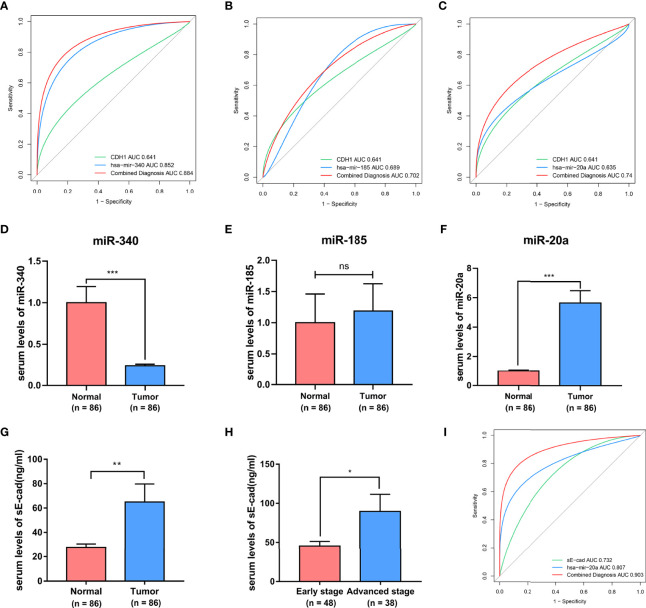
The diagnostic significances of sE-cad and miR-340, miR-185, miR-20a in BC. **(A–C)** The ROC curves for the diagnostic value of the CDH1, miR-340, miR-185, and miR-20a in BC. **(D–G)** Serum miR-340, miR-185, miR-20a, and sE-cad expression levels in BC patients and healthy subjects. **(H)** sE-cad in serum levels of BC patients at different stages. **(I)** ROC curves of sE-cad in combination with miR-20a diagnostic model. *P < 0.05; **P < 0.01; ***P < 0.001. no significant difference.

Previous bioinformatics indicated that CDH1, miR-340, miR-185, and miR-20a are overexpressed in BC and related to its malignant progression. ROC curve analysis was also used to investigate the diagnostic potential of CDH1, miR-340, miR-185 and miR-20a as biomarkers for BC. We discovered that combing CDH1 with miR-340, miR-185, or miR-20a could improve the diagnostic efficiency in BC, with areas under the ROC curve (AUC) of 0.884, 0.702, and 0.74, respectively (**Figures 6A–C**). Furthermore, to validate the clinical significance of the target gene as a non-invasive biomarker, we collected serum from BC patients and healthy people and detected the expression of CDH1 encoded E-cad secreted serum sE-cad, miR-340, miR-185, and miR-20a. Compared to the healthy control group, we discovered that miR-340 was significantly lower expressed in BC patients (**Figure 6D**), miR-185 had no significantly differential expression in BC patients (**Figure 6E**), miR-20a and sE-cad levels were significantly highly increased in BC patients (**Figures 6F, G**). Patients in advanced stage had significantly higher sE-cad expression than early stage (**Figure 6H**). Correlation analysis showed that high level of sE-cad in serum was positively associated with stage (*P* = 0.036), lymphoid nodal status (*P* = 0.005), and HER2 expression (*P* = 0.004) (**Table 2**). And it also indicated a positive association between increased miRNA-20a in serum and Ki67 expression (*P* = 0.001) (**Table 3**). Therefore, we assessed the efficacy of serum sE-cad, miR-20a, and the combination of sE-cad and miR-20a for diagnosing BC with AUCs 0.732, 0.807, and 0.903, respectively (**Figure 6I**). The findings suggest that miR-20a and sE-cad may be effective non-invasive biomarkers for BC diagnosis, and that their combination diagnosis can improve diagnostic efficiency.

**Table 2:** Correlations Between sE-cad in Sreum and Clinicopathological Parameters of Brest Cancer Patients.

		sE-cad expression		*P*-value
Variables	AllCases	Low (43)	High (43)
**Age**				
< 51	36	18	18	0.855
≥ 51	50	24	26	
**Stage**				
I+II	76	42	34	0.036*
III+IV	10	2	8	
**Lymphoid Nodal Status**				
Negative	44	28	16	0.005*
Positive	42	14	28	
**ER**				
Negative	36	14	22	0.117
Positive	50	28	22	
**PR**				
Negative	38	20	18	0.531
Positive	48	22	26	
**HER2**				
Negative	32	22	10	0.004*
Positive	54	20	34	
**Ki67**				
≤ 20	42	24	18	0.132
> 20	44	18	26	

*Significantly different.

ER, estrogen receptor; PR, progesterone receptor; HER2, human epidermal growth factor receptor 2; Ki-67, proliferating antigen Ki67.

*Bold values indicate P < 0.05.

**Table 3:** Correlations Between miRNA-20a in Sreum and Clinicopathological Parameters of Brest Cancer Patients.

Variables	Allcases	miRNA-20a expression		*P*-value
		Low (43)	High (43)
**Age**				
< 51	36	14	22	0.117
≥ 51	50	28	22	
**Stage**				
I+II	76	38	38	0.552
III+IV	10	4	6	
**Lymphoid Nodal Status**				
Negative	42	24	18	0.132
Positive	44	18	26	
**ER**				
Negative	36	16	20	0.489
Positive	50	26	24	
**PR**				
Negative	38	18	20	0.808
Positive	48	24	24	
**HER2**				
Negative	32	16	16	0.868
Positive	54	26	28	
**Ki67**				
≤ 20	42	28	14	0.001*
> 20	44	14	30	

*Significantly different.

ER, estrogen receptor; PR, progesterone receptor; HER2, human epidermal growth factor receptor 2; Ki-67, proliferating antigen Ki67.

*Bold values indicate P < 0.05.

## Discussion

Due to rapid increase in the incidence and mortality of BC in recent years, BC management has remained a major challenge. Various biomarkers for BC have been proposed, including CA153, CEA, P53, HER-2, ER, and PR (38, 39). However, it is difficult to predict the biological potential of BC using these biomarkers. Recent research has revealed that tumor stemness biomarkers can predict tumor progression and provide diagnostic and therapeutic strategies based on tumor stemness (36, 40). Screening for biomarkers associated with the stem cell signature of breast cancer is thus critical in improving the diagnosis and survival of BC patients.

CDH1 has previously been identified as a tumor suppressor gene. CDH1 gene mutation, DNA hypermethylation silencing, and its encoded E-cad dysfunction are all involved in the invasion and metastatic progression of numerous cancers (41–43). However, a small number of published studies have demonstrated that CDH1 has the pro-oncogenic activity. CDH1 upregulation can maintain the stemness characteristics of tumor cells and promote oncogenesis and progression (8, 13). In addition, many miRNAs participate in regulating cancer stem cells characteristics (44)and they play an important role in the diagnosis and prognosis of tumors as new, easily accessible, affordable, non-invasive biomarker (45). Prior to this study, the oncogenic activity and clinical significance of CDH1 and its related miRNAs in regulating BC function have not been thoroughly investigated and established. This study has made the following novel findings:

First, bioinformatic analysis of publicly available cancer databases (including TIMER 2.0, GEPIA, Oncomine, TCGA, GEO, HPA, and Kaplan-Meier plotter) revealed that highly expressed CDH1 functions as an oncogene in BC and is positively correlated with malignancy progression (**Figures 1**, **2**). Early studies suggest that CDH1, as a tumor suppressor gene, as evidenced by mutations or methylation of CDH1, silences CDH1 expression, thus increasing the incidence of BC, as well as infiltrative tumor growth and metastasis (46, 47). However, in the present study, CDH1 expression was found to be elevated in all BC subtypes, and CDH1 upregulation was positively related to BC patient stage, metastatic, poor prognosis, and stemness signature. The findings of our study suggest that CDH1 may play an oncogene role in tumorigenesis and development of BC. Meanwhile, CDH1 has been reported to be a stemness gene. Increased CDH1 expression in embryonic stem cells contributes to pluripotency maintenance and prevents cell differentiation (48). Ye et al. (13) demonstrated that CDH1 is essential for the self-renewal of lung cancer stem-like cells. These findings uncovered a novel mechanism understanding by which high CDH1 expression may act as an oncogene by regulating tumor stemness characteristics. However, further in-depth mechanistic characterization will answer whether CDH1 is a novel target for therapeutic development.

Second, miRNA target gene prediction and analysis revealed that enhanced miR-340, miR-185, and miR-20a could target CDH1 in BC. Among them, miR-20a overexpression was positively linked to the stemness characteristics and poor prognosis of BC, which might be the pro-oncogene associated with stemness progression of breast cancer. In fact, miR-340 has been widely reported as a tumor suppressor (49). In BC, miR-340 overexpression was shown to significantly inhibit BC cell migration and invasion (50, 51). However, our study discovered that, despite being highly expressed in BC, miR-340 did not correlate with either stemness characteristics or prognosis of BC. Low expressed miR-185 has been reported to contribute to the acquisition of stemness characteristics in BC cells when regulated by LINC00511 (52), as opposed to our prediction that high expression of miR-185 is positively associated with stemness characteristics in BC. The discrepancies exist between our findings and those on miR-340 and miR-185 as a tumor suppressor. More research is required to determine their role in BC progression. Furthermore, high miR-20a expression was positively associated with both stemness features and the poor prognosis of BC. miR-20a has been widely reported as an oncogenic miRNA, which confirms our prediction. In BC, miR-20a could promotes the proliferation and invasion of BC cells by targeting ZBTB4 or PTEN (53, 54). In addition, high expression of miR-20a upregulates the self-renewal and proliferation of gastric cancer stem cells and is positively correlated with the poor prognosis of gastric cancer (55, 56). It implies that miR-20a acts as an oncogenic molecule in BC and may contribute to malignant progression and poor prognosis by promoting tumor stemness features. To better understand the biogenesis of miR-20a regulated, we further investigate all the targets of miR-20a other than CDH1, and implemented HALLMARK, KEGG, GO enrichment to discovery the functions in which the miR-20a participated in the tumorigenesis and development (**Supplement 1**). Notably, the possible relation of miR-340, miR-185, and miR-20a to CDH1 and how miRNA regulates stemness in BC should be further revealed by experimental evidence.

Third, using bioinformatics analysis, this study innovatively predicted the potential signaling pathways underlying the oncogenic activity of high expression of CDH1 in combination with overexpression of miR-340, miR-185, or miR-20 in BC. Of these, GO, and GSEA enrichment analyses showed that miR-340 or miR-185 combined with CDH1 overexpression existed in common enrichment pathways, such as glycolysis, MTORC1 signaling, steroid biosynthesis, peroxidase, and ubiquitination binding, which are closely related to cancer cell metabolism. It is well known that altered metabolism is one of the hallmarks of cancer. Numerous cancer cells rely on aerobic glycolysis for nutrients and energy (57).The activation of SREBP1 by mTORC1 in BC cells inhibits adipogenesis and interferes with cancer cell proliferation and tumor growth (58). Additionally, BC is a malignancy in which steroid hormones drive cellular proliferation, such as the sex steroid hormones estrogen receptor (ER) and progesterone receptor (PR), which are important prognostic and predictive markers for BC (59). Although miR-340 and miR-185 have been reported as tumor suppressor miRNAs, the mechanism by which miR-340 and miR-185 targeting CDH1 has not been investigated, and further studies are required to determine the oncogenic regulatory mechanism. Of note, CDH1 co-overexpression in combination with miR-20a was highly enriched mainly in histone methyltransferase activity, MYC targets V2, WNT/BETA-CATENIN signaling and ribosomes. Among them, MYC and WNT/BETA-CATENIN signaling pathways are important regulatory pathways for cancer stem cell self-renewal (4, 60). Recent studies have reported that histone methyltransferase EZH2 plays a critical role in maintaining ovarian CSC stemness (61). Glioma cells acquire stem-like characters by extrinsic ribosome stimuli (62). This indicates that the potential oncogenic mechanism of miR-20a-targeted regulation of CDH1 may be intricately linked with the stemness progression of BC, which warrants further investigation.

Fourth, we provide convincing experimental evidence supporting that sE-cad, which is formed by the secretion of CDH1-encoded E-cad into serum and combined with miR-20a detection, has better diagnostic potential in BC. In a recent study, the screening for biomarkers associated with cancer stem cell signatures provided novel insights into the selection of tumor diagnostic biomarkers (36, 63). Consequently, we further explored the potential of CDH1 and miR-340, miR-185 and miR-20a as non-invasive diagnostic markers for BC patients. Our results show that sE-cad was significantly highly expressed in the sera of BC patients and positively related to the BC stage and lymphoid nodal status, consistent with bioinformatics analysis results that CDH1 overexpression was positively linked to malignant progression of BC. According to literature, miR-20a has a good diagnostic value in colorectal cancer (AUC=0.70) (64). Our study showed that miR-20a had comparatively good diagnostic potential in BC, with an AUC of 0.807. Furthermore, sE-cad combined with miR-20a assay improved BC diagnostic efficiency with an AUC of 0.903.

In conclusion, this study provides evidence for CDH1 as an oncogene in BC and suggests that miR-20a may regulate the stemness characteristics of BC to exert a pro-oncogenic effect by regulating CDH1. Simultaneously, sE-cad and miR-20a in serum can both be used as valid noninvasive markers for BC diagnosis. Therefore, these results demonstrate the potential of CDH1 and its related miRNAs as new clinical targets for the diagnosis, prognosis and treatment of BC which will be explored in our future studies.

## Data Availability Statement

The datasets presented in this study can be found in online repositories. The names of the repository/repositories and accession number(s) can be found in the article/**Supplementary Material**.

## Ethics Statement

The studies involving human participants were reviewed and approved by the Ethical Committee of the Affiliated Hospital of Southwest Medical University. The patients/participants provided their written informed consent to participate in this study.

## Author Contributions

The concept and design of the present study were mainly provided by TY. DX, YC, and XW contributed to drafting and editing the manuscript. Experiment data were collected and analyzed by QP, DX, and YL. JYL, JBL, YC, and XW performed the data acquisition and data analysis. Finally, TY conducted data auditing and manuscript review. All authors contributed to the article and approved the submitted version.

## Funding

This work was supported by the National Natural Science Youth Fund, China (Grant No.82003138), the Sichuan Science and Technology Program for key Research and Development, China (Grant No.2021YFS0226), Science and technology strategic cooperation project of Luzhou People’s Government and Southwest Medical University (Grant No.2021LZXNYD-J08), Doctoral Research Initiation Fund of Affiliated Hospital of Southwest Medical University, China (Grant No.19077) and University’s scientific research project of Southwest Medical University, China (Grant No. 2020ZRQNA019).

## Conflict of Interest

The authors declare that the research was conducted in the absence of any commercial or financial relationships that could be construed as a potential conflict of interest.

## Publisher’s Note

All claims expressed in this article are solely those of the authors and do not necessarily represent those of their affiliated organizations, or those of the publisher, the editors and the reviewers. Any product that may be evaluated in this article, or claim that may be made by its manufacturer, is not guaranteed or endorsed by the publisher.

## Supplementary Material

Supplement 1 The roles of miR-20a in BC. **(A)** Differential expressions of miR-20a in I+II and III+IV tumor stage in BC. **(B)** Venn diagram of miR-20a predicted to regulate potential targets from a total of four databases: Targetscan7, miRDB, miRWalk, and starBase. **(C)** The potential targets of miR-20a. **(D)** Hallmark gene set enrichment analysis of miR-20a in BC. **(E)** KEGG pathways enrichment analysis of miR-20a in BC. **(F)** Molecular functions enrichment analysis of miR-20a in BC. **(G)** Biological process enrichment analysis of miR-20a in BC.
