# Supplementary figures and images for: The Potential Role of CDH1 as an Oncogene Combined With Related miRNAs and Their Diagnostic Value in Breast Cancer
Source: Front Endocrinol (Lausanne). 2022 Jun 16;13:916469. doi: 10.3389/fendo.2022.916469 (PMC9243438; doi:10.3389/fendo.2022.916469)

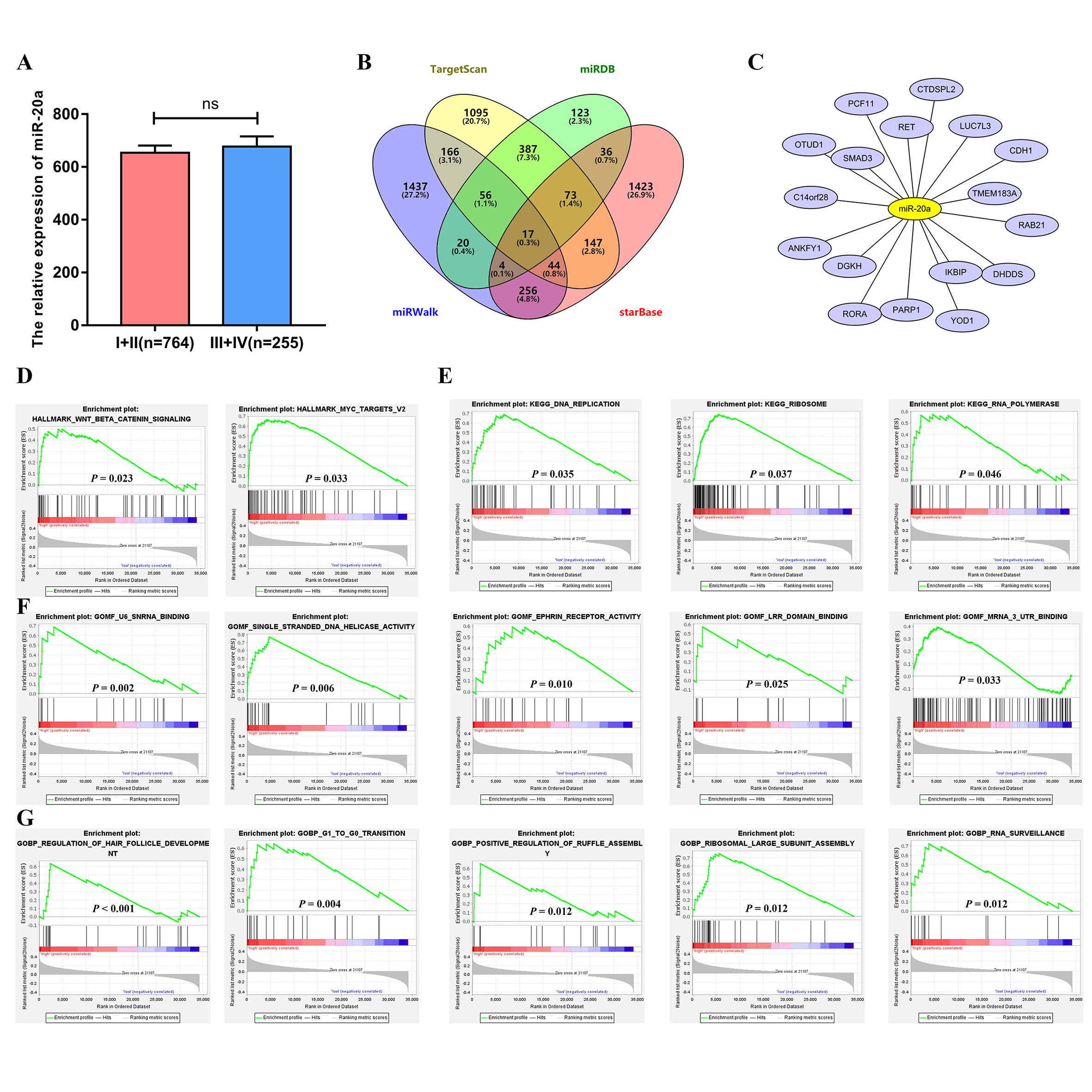

Supplement: Supplement 1 — The roles of miR-20a in BC. (A) Differential expressions of miR-20a in I+II and III+IV tumor stage in BC. (B) Venn diagram of miR-20a predicted to regulate potential targets from a total of four databases: Targetscan7, miRDB, miRWalk, and starBase. (C) The potential targets of miR-20a. (D) Hallmark gene set enrichment analysis of miR-20a in BC. (E) KEGG pathways enrichment analysis of miR-20a in BC. (F) Molecular functions enrichment analysis of miR-20a in BC. (G) Biological process enrichment analysis of miR-20a in BC. [file Image_1.jpeg]
